# Insights into Cadmium-Induced Carcinogenesis through an In Vitro Study Using C3H10T1/2Cl8 Cells: The Multifaceted Role of Mitochondria

**DOI:** 10.3390/ijms221910837

**Published:** 2021-10-07

**Authors:** Monica Oldani, Anna Maria Villa, Marta Manzoni, Pasquale Melchioretto, Paolo Parenti, Eugenio Monti, Paola Fusi, Matilde Forcella, Chiara Urani

**Affiliations:** 1 Department of Biotechnology and Biosciences, University of Milano-Bicocca, Piazza della Scienza 2, 20126 Milano, Italy; m.oldani12@campus.unimib.it (M.O.); annamaria.villa@unimib.it (A.M.V.); 2 Department of Molecular and Translational Medicine (DMTM), University of Brescia, Viale Europa 11, 25123 Brescia, Italy; marta.manzoni@unibs.it (M.M.); eugenio.monti@unibs.it (E.M.); 3 Department of Earth and Environmental Sciences, University of Milan Bicocca, Piazza della Scienza 1, 20126 Milan, Italy; pasquale.melchioretto@unimib.it (P.M.); paolo.parenti@unimib.it (P.P.); chiara.urani@unimib.it (C.U.); 4 Integrated Models for Prevention and Protection in Environmental and Occupational Health, (MISTRAL), Interuniversity Research Center, 37134 Verona, Italy

**Keywords:** cadmium, cancer, metabolism, mitochondria, transformed *foci*

## Abstract

In this paper, we report the metabolic characterization of two *foci*, F1 and F3, obtained at the end of Cell Transformation Assay (CTA), performed by treating C3H10T1/2Cl8 mouse embryo fibroblasts with 1 μM CdCl_2_ for 24 h. The elucidation of the cadmium action mechanism can be useful both to improve the in vitro CTA and to yield insights into carcinogenesis. The metabolism of the two *foci* was investigated through Seahorse and enzyme activity assays; mitochondria were studied in confocal microscopy and reactive oxygen species were detected by flow cytometry. The results showed that F1 *focus* has higher glycolytic and TCA fluxes compared to F3 *focus*, and a more negative mitochondrial membrane potential, so that most ATP synthesis is performed through oxidative phosphorylation. Confocal microscopy showed mitochondria crowded in the perinuclear region. On the other hand, F3 *focus* showed lower metabolic rates, with ATP mainly produced by glycolysis and damaged mitochondria. Overall, our results showed that cadmium treatment induced lasting metabolic alterations in both *foci*. Triggered by the loss of the Pasteur effect in F1 *focus* and by mitochondrial impairment in F3 *focus*, these alterations lead to a loss of coordination among glycolysis, TCA and oxidative phosphorylation, which leads to malignant transformation.

## 1. Introduction

Cadmium (Cd) is a well-known carcinogen, classified by the International Agency for Research on Cancer as a group I human carcinogen; however, the molecular mechanisms leading cells into a malignant phenotype are largely unknown. Still, cadmium is a widespread environmental contaminant, currently released by anthropogenic activities at a rate of 30,000 tons per year. Human uptake is therefore very easy: besides occupational exposure, it can occur through food, drinking water, inhalation of air particles, and cigarette smoking. The absence of any specific excretory way for cadmium leads to its persistence inside the body, with a half-life between 10 and 30 years [1].

With the exception of a catalytic role in some algal enzymes [2], cadmium is devoid of all biological roles; however, Cd^2+^ ions can easily displace Zn^2+^ ions, due to their similar charge and mass [3,4,5,6]. This similarity also accounts for cadmium uptake by zinc channels and transporters, in what has been named a “Trojan horse mechanism” [7]. Moreover, cadmium can interfere with zinc binding proteins, whose estimated number within the cell is higher than 3,000. These features lead to a major displacement of Zn in the Zn-proteome, and the substitution with Cd in the Zn-finger regions. Consequences of these chemical properties are the disruption of the correct folding and the inactivation of proteins involved in genomic stability, such as the p53, and of proteins involved in DNA repair (e.g., NER and BER) [6,8].

Cell Transformation Assay (CTA), the most advanced in vitro test for the prediction of human chemical carcinogenicity, refers to the induction, in cultured cells, of phenotypic alterations that have long been considered associated with cells exhibiting neoplastic potential in vivo. After exposure to carcinogenic stimuli, such cells show morphological alterations and form discrete anchorage-independent altered colonies, referred to as transformed *foci*, atop the confluent monolayer. The transformed *foci* are able to produce tumors in vivo. Moreover, the CTA has been shown to closely model some key stages of the in vivo carcinogenic process [9]. In addition to be widely used for the screening of potential carcinogens [10,11], the CTA is also a powerful tool for mechanistic studies of carcinogenesis [12,13]. In our experiments, the CTA based on the use of C3H10T1/2Cl8 mouse embryo fibroblasts was adopted, the latter being among the suitable cells suggested by standard protocols [14].

*Foci*, obtained at the end of the CTA, are recognized under a microscope and classified by morphological features, such as deep basophilic staining, multilayered growth, random cell orientation at the edge of the focus, and invasiveness of the surrounding normal cells monolayer [9,14]. These morphological features are related to molecular changes leading the cells to acquire fully malignant characteristics, which are demonstrated by their ability to develop tumors when injected into susceptible host animals [15].

In a previous work [16], we found that morphological evaluations and proliferative assays confirmed the loss of contact inhibition and the higher proliferative rate of transformed clones. Moreover, biochemical analysis of EGFR pathway revealed that, despite the same initial carcinogenic stimulus (1 μM CdCl_2_ for 24 h), the different *foci* were characterized by the activation of different molecular pathways. In particular, F1 *focus* showed ERK activation and a high proliferation rate, while F3 *focus* showed Akt activation and a survival molecular profile. More recently [12], a toxicogenomic study performed by our group, showed that, upon cadmium administration, the two *foci* also developed distinct patterns of up-regulated and down-regulated genes.

This work reports an in-depth characterization of both F1 and F3 *foci*, with the aim of identifying metabolic alterations caused by gene dysregulation. We investigated oxygen consumption rate and ATP production, as well as mitochondrial morphology and defense mechanisms against oxidative stress; mitochondria are in fact known to play a key role in malignant transformation and metastasis development [17]. Our results show a different metabolic pattern in each *focus*, which could explain their different proliferation rates.

## 2. Results

### 2.1. The Main ATP Production Route Is Oxidative Phosphorylation in F1 Focus and Substrate Level Phosphorylation in F3 Focus

**Figure 1:**
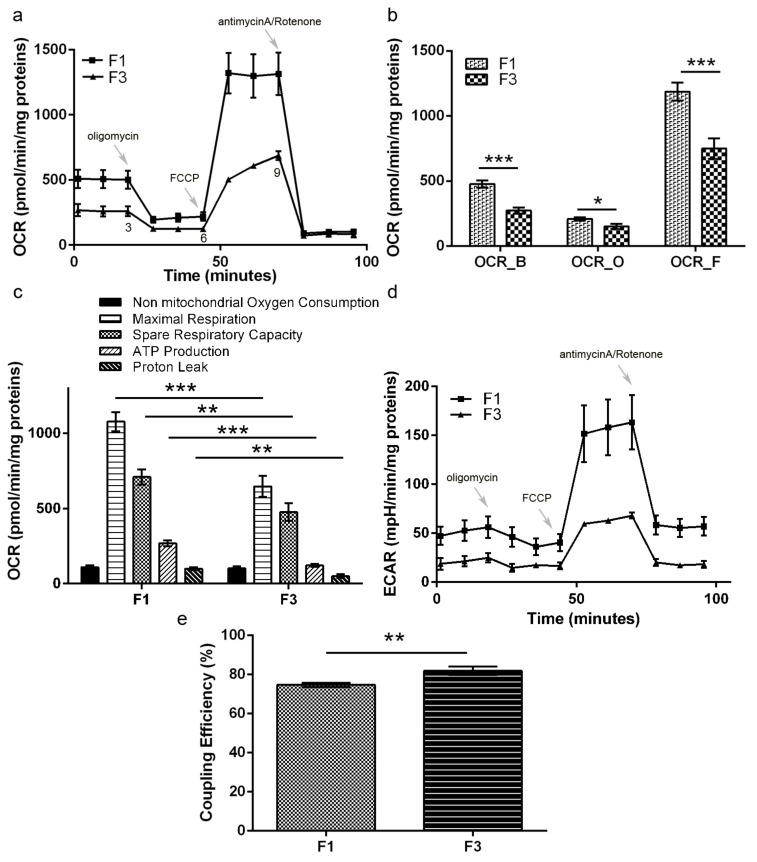
Seahorse Mitostress analysis. (**a**) OCR traces, expressed as pmoles O_2_/min/mg proteins in F1 and F3 *foci*. The arrows indicate the addition time of oligomycin, FCCP and antimycin A/rotenone. The OCR profile is representative of three independent experiments. (**b**) The values at points 3, 6, 9 of OCR profile in panel (**a**) reflect OCR_B (basal), OCR_O (oligomycin) and OCR_F (FCCP). (**c**) Analysis of different parameters related with mitochondrial function. (**d**) ECAR traces, expressed as mpH/min/mg proteins, in F1 and F3 *foci*. The ECAR profile is representative of three independent experiments. (**e**) Coupling efficiency. Bars indicate the mean ± SEM obtained in three independent experiments. Statistical significance: * *p* < 0.05, ** *p* < 0.01, *** *p* < 0.001 (Student’s *t*-test).

The respiratory metabolism of both F1 and F3 *foci* was investigated, measuring oxygen consumption rate (OCR) and extracellular acidification rate (ECAR), through Mitostress assay in the Agilent Seahorse. The results are reported in Figure 1: F1 *focus* showed higher basal and maximal respiration rates, as well as a higher spare respiratory capacity, compared to F3 *focus* (Figure 1a,b). The higher basal OCR shown by F1 *focus* is reflected by the higher level of acidification, as shown by ECAR measurement, reported in Figure 1d. Both F1 and F3 ECAR was only slightly decreased upon oligomycin addition, while it increased following FCCP addition; however, the F1 ECAR increase was found to be much higher than F3 and remained high even after rotenone/antimycin addition, suggesting a significant contribution of glycolytic acidification. As shown in Figure 1c, the proton leak, measuring the amount of protons which are not used as proton motive force, was also found to be more consistent in F1 *focus*. As a consequence, in this focus electron transport is less efficiently coupled to ATP synthesis (20% uncoupling), as reported in Figure 1e. Despite mitochondria uncoupling, F1 higher basal respiration rate sustained a higher mitochondrial ATP production, compared to F3 *focus* (Figure 1c).

**Figure 2:**
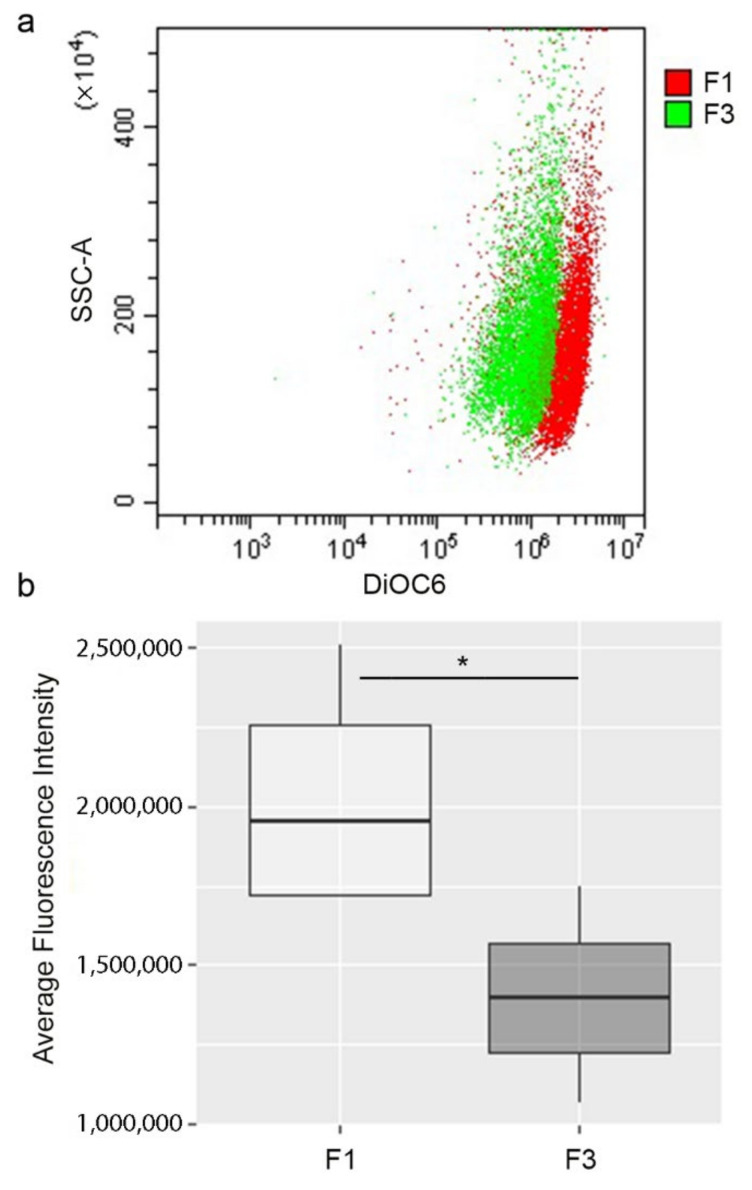
Flow cytometry analysis of Δψ in F1 and F3 *foci*. (**a**) After incubation with DiOC6 the level of cells fluorescence was measured. The results are shown in a dot plot overlay, representative of three independent experiments. (**b**) The fluorescence intensity of all experiments is represented by a box plot. The dark line within the box represents the median value, while the upper and lower sides of the box are the third and first quartiles, respectively. Statistical significance: * *p* < 0.05 (Student’s *t*-test).

Mitochondrial membrane potential was measured through the green-fluorescent, lipophilic dye 3,3′-dihexyloxacarbocyanine iodide (DiOC6). As reported in Figure 2, in accordance with its higher mitochondrial ATP production and higher OCR, F1 *focus* showed a higher DiOC6 fluorescence, compared to F3 *focus*, indicating a more negative Δψ.

### 2.2. F1 Focus Shows Hyperactivated Glycolysis, TCA and Lactic Fermentation

The activities of a series of enzymes involved in sugar metabolism were assayed in both *foci*. The results are reported in Table 1: glyceraldehyde-3-phosphate dehydrogenase (GAPDH) was found to be more active in F1 *focus* than in F3; hexokinase (HK) and pyruvate kinase (PK), although showing a modest difference between the two *foci*, were also found reduced in F3 *focus*; moreover, both citrate synthase and malate dehydrogenase specific activities were significantly higher in F1 *focus*.

**Figure 3:**
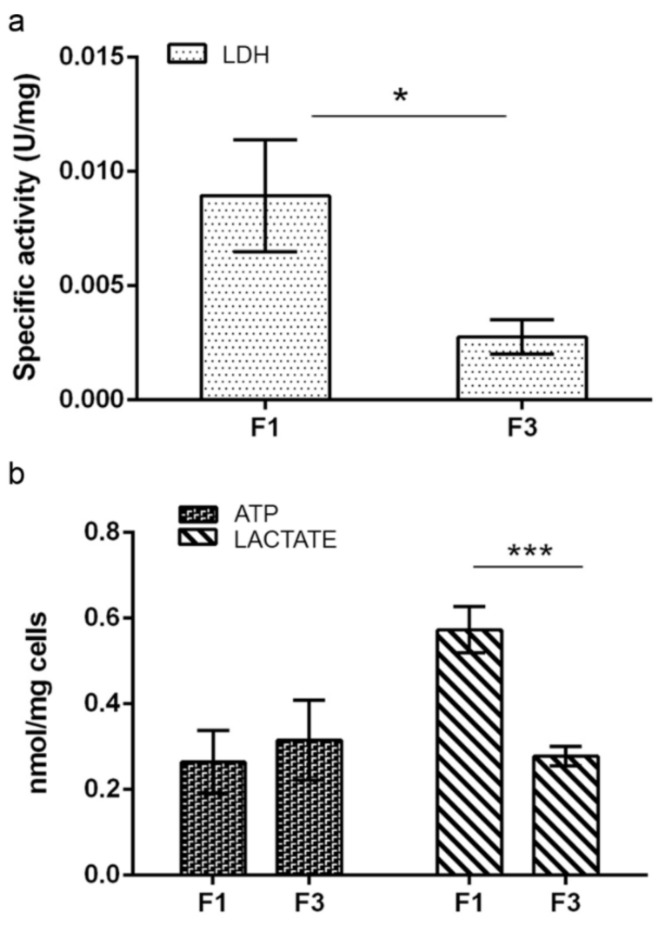
(**a**) Lactate dehydrogenase activity analysis in F1 and F3 *foci*. Results are expressed as U/mg. (**b**) ATP and lactate level in F1 and F3 *foci*. Results are expressed in nmol and normalized with respect to mg of cells. All results are shown as mean ± SEM obtained in three independent experiments. Statistical significance: * *p* < 0.05, *** *p* < 0.001 (Student’s *t*-test).

On the contrary, a significant increase in glutamate dehydrogenase activity was detected in F3, compared to F1 *focus*, while no differences between the two *foci* were observed in specific activities of malic enzyme, glucose-6-phosphate dehydrogenase (G6PDH) and NADP^+^-dependent isocitrate dehydrogenase. As shown in Figure 3a, lactate dehydrogenase (LDH) was also found more active in F1 *focus*, suggesting that glycolytic NADH production exceeds mitochondrial reoxidative capacity leading to fermentation. This is confirmed by the increase in lactate production, shown by F1 *focus* (Figure 3b).

**Figure 4:**
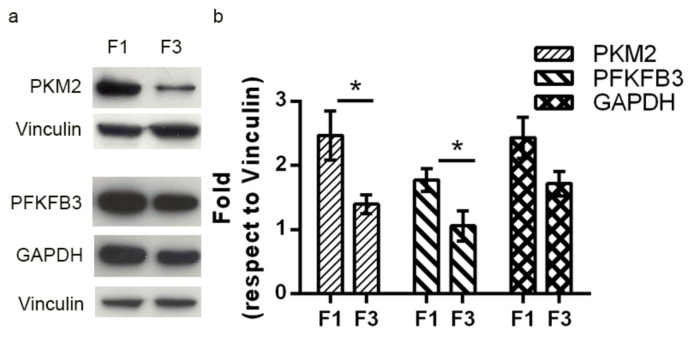
(**a**) Representative Western-blot analysis performed on crude extracts, using anti-PKM2, anti-PFKFB3 and anti-GAPDH antibodies. Vinculin was used as loading control. The experiments were performed in triplicate. (**b**) Densitometric analysis was performed with Scion Image Software. Values are presented as means ± SEM. Statistical significance: * *p* < 0.05 (Student’s *t*-test).

To assess whether F1 *focus* metabolism is rewired into the Warburg effect, we investigated the expression of PKM2 isoform of pyruvate kinase, which is often expressed in cancer cells, as well as the expression of the PFKFB3 isoform of the bifunctional phosphofructokinase/fructose bisphosphatase (PFK/FBP) enzyme. PFKFB3 is endowed with a much higher kinase/phosphatase activity, compared to the normal enzyme, and allows cancer cells to maintain high glycolytic rates, by producing fructose 2,6-bisphosphate; the latter in turn activates phosphofructokinase activity of the bifunctional PFK1/FBP enzyme making it at the same time insensitive to ATP inhibition. As reported in Figure 4, Western blots showed that PKM2 was expressed in both foci, although at higher levels in F1 focus. This is well in accordance with a higher ATP production in F1 *focus* through oxidative phosphorylation than through glycolysis: in fact, PKM2 isoform is less active and allows the PEP phosphate group to be transferred to the active site of phosphoglyceromutase. This results in the net conversion of PEP into pyruvate, without ATP production.

The PFKFB3 isoform and glyceraldehyde-3-phosphate dehydrogenase (GAPDH) were also found to be expressed at a higher level in F1 *focus* than in F3 *focus*, accounting for F1 higher glycolytic flux.

### 2.3. F3 Focus Shows Impaired Oxidative Phosphorylation

**Figure 5:**
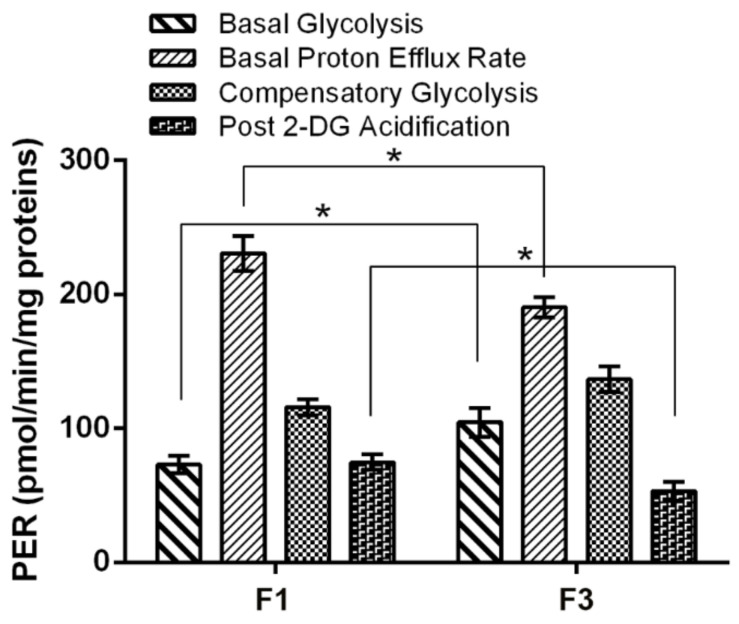
Seahorse glycolytic analysis in F1 and F3 *foci*. Analysis of different parameters related with glycolysis. Bars indicate the mean ± SEM obtained in three independent experiments. Statistical significance: * *p* < 0.05 (Student’s *t*-test).

Although F1 *focus* showed a higher mitochondrial ATP production, total intracellular ATP was found to be the same in both *foci*, as shown in Figure 3b, suggesting that F3 *focus* compensated for this with a higher ATP generation through substrate level phosphorylation. Both glycolysis and oxidative phosphorylation are less active in F3 *focus*, in accordance with its reduced proliferation, compared to F1, leading to a minor consume of ATP. This was confirmed by OCR and ECAR measurements through Seahorse glycolytic assay, reported in Figure 5; the Proton Efflux Rate (PER) was measured for each *focus* under different conditions. The results showed that, although basal PER was higher for F1 *focus*, F3 *focus* showed a higher basal glycolysis, as well as a higher compensatory glycolysis. Moreover, the acidification level dropped to lower values after glycolysis inhibition by 2-DG, confirming that F3 *focus* relies more on substrate level phosphorylation than on oxidative phosphorylation. Further confirmation comes from specific activity assays of glycolytic and TCA enzymes, reported in Table 1, showing that, while some TCA enzymes (such as pyruvate kinase, citrate synthase and malate dehydrogenase) were found to be less active in F3 *focus*; compared to F1, glutamate dehydrogenase activity was found to be significantly higher; this suggests that F3 *focus* may use TCA in an anaplerotic way.

### 2.4. F3 Focus Generates More ROS, but Less O_2_^−^, Compared to F1 Focus

**Figure 6:**
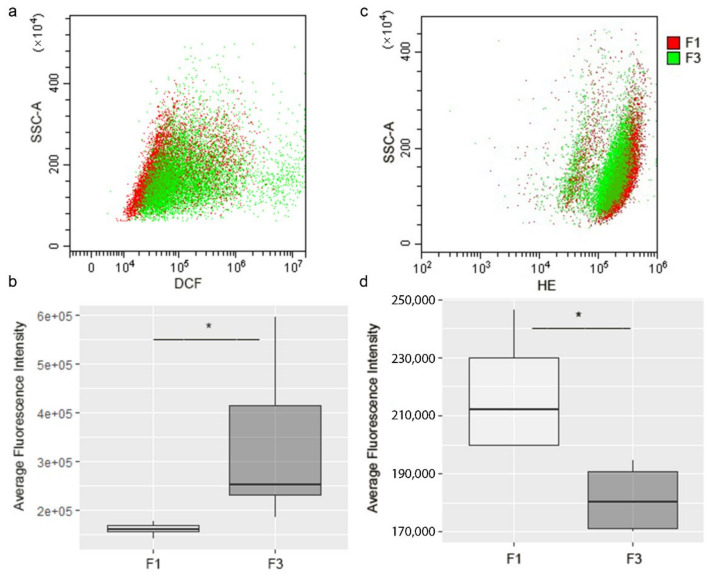
Flow cytometry analysis of ROS and superoxide anion production in F1 and F3 *foci*. After incubation with H_2_DCFDA or DHE the level of cells fluorescence was measured. Results are shown in a dot plot overlay, representative of three independent experiments (**a**–**c**). The fluorescence intensity of all experiments is represented by a box plot (**b**–**d**). The dark line within the box represents the median value, while the upper and lower sides of the box are the third and first quartiles, respectively. Statistical significance: * *p* < 0.05 (Student’s *t*-test).

Total ROS content was assayed in each focus through cell-permeant 2′,7′-dichlorodihydrofluorescein diacetate (H_2_DCFDA), which is oxidized to DCF fluorescent probe; results, reported in Figure 6a,b, showed a higher ROS level in F3 *focus*. However, DHE fluorescent probe, more selective towards O_2_^−^, showed that this ROS was more abundant in F1 *focus*, as reported in Figure 6c,d.

**Figure 7:**
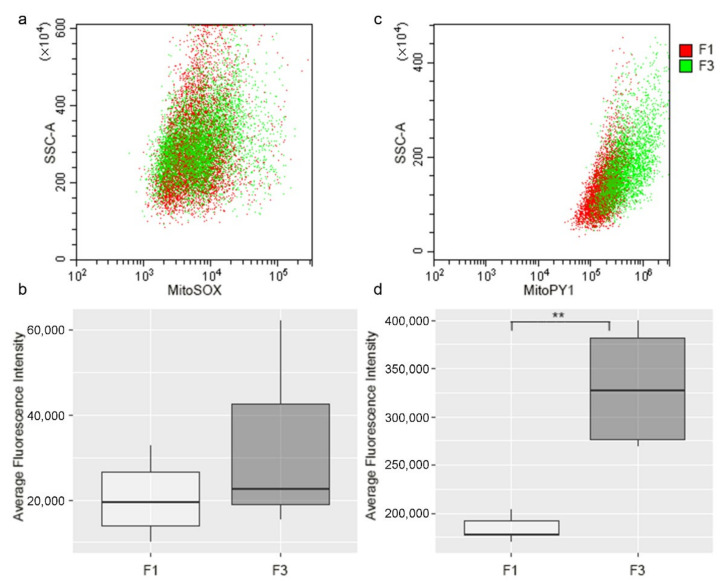
Flow cytometry analysis of mitochondrial superoxide anion and H_2_O_2_ production in F1 and F3 *foci*. After incubation with MitoSOX ™ Red or MitoPY1, the level of cells fluorescence was measured. Results are shown in a dot plot overlay, representative of three independent experiments (**a**–**c**). The fluorescence intensity of all experiments is represented by a box plot (**b**–**d**). The dark line within a box represents the median value, while the upper and lower sides of the boxes are the third and first quartiles, respectively. Statistical significance: ** *p* < 0.01 (Student’s *t*-test).

This is confirmed by investigation of mitochondrial ROS generation, using MitoSOX Red indicator, which reacts with superoxide, and by MitoPY1, detecting hydrogen peroxide; results showed no significant difference in mitochondrial O_2_^−^ production between F1 and F3 *foci* (Figure 7a,b), while H_2_O_2_ production was found to be more elevated in F3 *focus* (Figure 7c,d).

**Figure 8:**
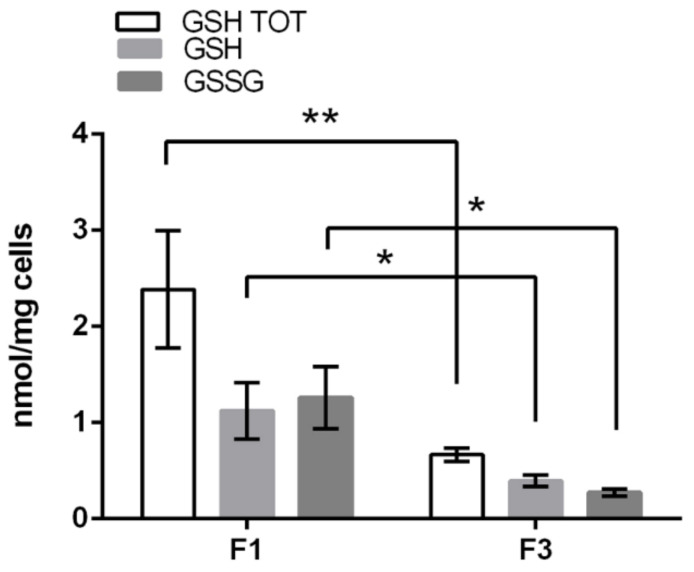
Glutathione level in F1 and F3 *foci*. Results are expressed in nmol/mg and normalized with respect to mg of cells. Statistical significance: * *p* < 0.05, ** *p* < 0.01 (Student’s *t*-test).

These results were further confirmed by the activity assays of the enzymes involved in oxidative stress defense, reported in Table 2. Glutathione S-transferase and glutathione reductase specific activities were both found higher in F1 *focus* than in F3 *focus*, while glutathione peroxidase and catalase specific activities were more elevated in F3 *focus*. The level of SOD1 and SOD2 were similar in both *foci*. Glutathione assay showed that total glutathione level was significantly higher in F1 *focus* (Figure 8), with a GSH/GSSG close to 1 for both *foci*.

### 2.5. Mitochondria Morphology Analysis Shows Different Alterations in Each Focus

**Figure 9:**
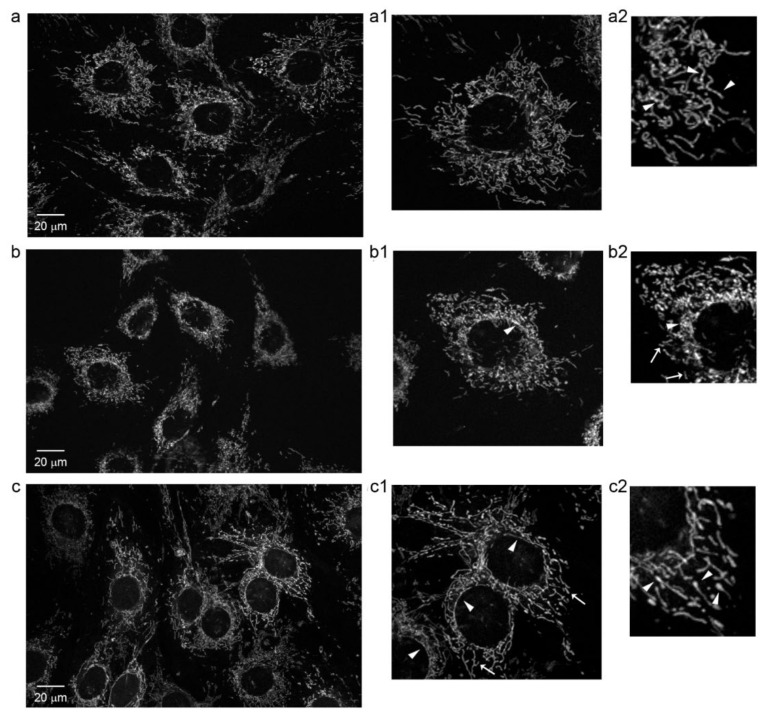
Representative mitochondrial confocal images of: (**a**) Healthy C3H10T1/2Cl8 cells; enlarged views: (**a1**) thin and filamentous mitochondria are evenly distributed in the whole cytoplasm; (**a2**) fluorescence is not uniform along single mitochondria showing regions with higher intensity (arrowheads). (**b**) *Focus* F1 cells; enlarged views: (**b1**) crowded mitochondria in the perinuclear region (arrowheads); (**b2**) short fragmented organelles are observed in the cytoplasm (arrows), where patches of crowded mitochondria are also seen (arrowheads). (**c**) *Focus* F3 cells; enlarged views: (**c1**) mitochondria are organized in an irregular network (arrows) and long filamentous mitochondria surround the cell nucleus (arrowheads); (**c2**) mitochondria appear thicker than those in control cells. The tips of mitochondria are often very fluorescent and swollen (arrowheads).

Confocal microscopy images of R123 stained cells from F1 and F3 *foci* were collected to investigate mitochondria morphology and intracellular distribution. The results are shown in Figure 9, together with images from healthy C3H cells, already described in a previous paper. As previously reported [18], mitochondria in C3H cells were found to be evenly distributed through the cytoplasm, extending from the nucleus to the cell periphery in a regular network (Figure 9a); their morphology was thin and filamentous (Figure 9a1), and their fluorescence intensity varied along the single mitochondrion, alternating regions of high and low fluorescence (Figure 9a2).

A different distribution and morphology were observed in F1 *focus* cells (Figure 9b), which showed tightly crowded mitochondria in the perinuclear region (Figure 9b1,b2). This mitochondrial crowding around the nucleus did not allow us to observe the detail of their morphology. At the cell periphery, patches of crowded mitochondria and rod-like shaped mitochondria were clearly observed, with short-fragmented organelles distributed in the whole cytoplasm (Figure 9b1,b2).

A different picture was seen for F3 *focus* (Figure 9c). In the cytoplasm, sparse mitochondria were organized in an irregular network and no crowding was observed in the perinuclear region, where few long filamentous mitochondria surrounded the cell nucleus. Mitochondria in these cells appeared to be generally thicker and longer than those in control cells (Figure 9c1,c2). A typical feature of these cells was the presence of mitochondria with very fluorescent and swollen tips (Figure 9c2), suggesting mitochondrial stress and a possible damage.

A quantitative analysis of mitochondria circularity and density of mitochondrial network, shown in Table 3, confirmed morphological data.

### 2.6. Autophagy Is Not Activated in Foci

**Figure 10:**
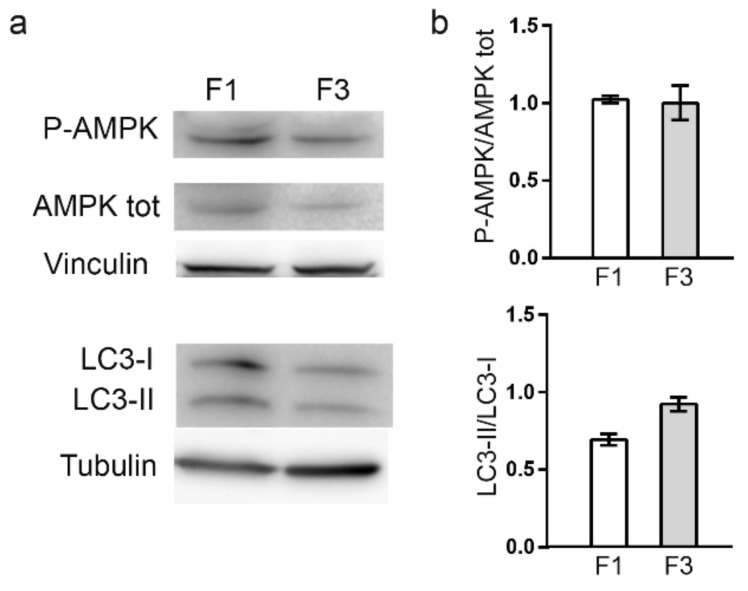
Autophagy evaluation in F1 and F3 *foci*. (**a**) Representative Western blot analysis performed on crude extracts, using anti-P-AMPK, anti-AMPK tot and anti-LC3B antibodies. Tubulin and Vinculin were used as loading control. (**b**) Densitometric analysis was performed with Scion Image Software. The results are expressed as P-AMPK/AMPK tot and LC3II/LC3I ratio and are shown as mean ± SEM from three independent experiments.

Autophagy activation was also investigated, since previous data, obtained in our laboratory, showed that a 24 h treatment with CdCl_2_ can induce autophagy in C3H10T1/2Cl8 mouse embryonic fibroblasts (Oldani et al. 2020b); however, neither F1 nor F3 *focus* showed autophagy activation, as reported in Figure 10, showing no difference in AMPK phosphorylation. The LC3-I and LC3-II isoforms’ distribution was also assessed and the amount of LC3-II, the isoform associated to autophagosomes, was compared between the two *foci*, in order to evaluate autophagy induction [19]; no significant differences were observed between the two *foci*.

## 3. Discussion

In this work we compared two different *foci*, F1 and F3, obtained at the end of the CTA performed on C3H cells treated with CdCl_2_ for 24 h. The results showed that previously observed differences in their proliferative behavior [16] and gene dysregulation [12] are accompanied by metabolic differences. This in turn confirms that there are many targets for cadmium at the molecular level, eventually leading to malignant transformation.

In F1 *focus*, we observed higher glycolytic, TCA and oxidative phosphorylation rates compared to F3 *focus*, which are well in accordance with its higher proliferative rate supported by increased mitochondrial functionality. This is likely accomplished through a mitochondrial reorganization in a tightly crowded distribution around the nucleus, as shown by confocal microscopy analysis. A similar picture was previously observed in C3H cells treated with CdCl_2_ for 24 h [18]. However, all the NADH produced by Cd-treated C3H10T1/2Cl8 cells was efficiently reoxidized on the electron transport chain. In F1 *focus*, NADH production exceeds mitochondrial reoxidative capacity, so that NADH is partly reoxidized by LDH, producing lactate. Another interesting feature of F1 *focus* is the loss of the Pasteur effect: although most ATP is produced through oxidative phosphorylation, glycolysis is not inhibited. This is likely due to the overexpression of PFKFB3, which, unlike PFK1FBP, is not inhibited by ATP and citrate [20] and is endowed with a much higher ratio in kinase/phosphatase activity, allowing cells to maintain high glycolytic rates. F1 high glycolytic rate could also sustain other metabolic synthesis, through overexpression of PKM2 isoform, promoting PEP conversion into pyruvate without ATP synthesis. This could lead to the synthesis of many metabolites, essential to sustain high proliferation rates, and particularly to glutathione synthesis, which is in fact more abundant in F1 *focus* compared to F3. Glutamine is also required for glutathione synthesis and further experiments will assess whether this amino acid contributes to F1 high proliferation.

F3 *focus* relies mainly on glycolysis for its ATP request, although this pathway is on the whole less active than in F1 *focus*; NADH produced in the glycolytic pathway is reoxidized by LDH, but lactate production is lower than in F1 *focus*. The metabolic flux through glycolysis is reduced (as shown by lower PFKFB3, PKM2 and GAPDH expression), compared to F1 *focus*, justifying F3 lower proliferative rate.

F3 *focus* showed a higher accumulation of total ROS, with an increased production of mitochondrial H2O2, compared to F1 *focus*: this is well in accordance with higher specific activities of both glutathione peroxidase and catalase in F3 *focus*. ROS accumulation is also supported by the GSH/GSSG ratio close to 1, observed in both *foci*, while in healthy cells it normally ranges between 200:1 and 30:1 [21]. In particular, the increase in H_2_O_2_ production can be due to superoxide dismutation, reflecting in an increase in superoxide production in F3 *focus* as well. However, it could also be due to electron transfer impairment in F3 damaged mitochondria, as supported by confocal microscopy data and confirmed by Seahorse results. Mitochondrial damage is also supported by previous toxicogenomic data [12] obtained in our laboratory showing that 13 out of the 15 top up-regulated genes in F3 *focus* are involved in an interferon mediated inflammatory response, similar to that triggered by viral DNA and RNA [22]. Furthermore, the upregulation, in F3 *focus*, of TLR8 gene and IL-6 coding gene [12] suggests that mtRNA release from damaged mitochondria could be responsible for the observed inflammatory response. A similar result was previously observed in human monocytes, where viral RNA was detected by TLR8 resulting in IL-6 and TNF secretion [23]. The importance of inflammation in the process of carcinogenesis is widely recognized [24], indicating that damaged mitochondria-mediated inflammation could be one possible route to transformation. Overall, our data show that, although total ATP level is the same in both *foci*, F3 consumes less ATP, due to its low proliferation. Therefore, although glycolysis is less active in F3 *focus* than in F1, it is still sufficient to support its growth.

F1 *focus* showed a lower level of total ROS, but a higher production of O_2_^−^ compared to F3 *focus*, although no significant differences were found in mitochondrial O_2_^−^ levels. This is in accordance with the higher glutathione S-transferase and glutathione reductase specific activities detected in F1 *focus*, representing the defense against O_2_^−^ accumulation, and is further confirmed by the higher level of reduced glutathione in this *focus*; a GSH/GSSG ratio close to 1 suggests that oxidative stress is high in this *focus*. The glutathione level is often increased in cancer cells, as a result of increased oxidative stress and glycolysis up-regulation, where it leads to faster growth rates and resistance to a number of chemotherapeutic agents [25]. Additionally, GSH has been shown to directly reduce O_2_^−^ [26] and may therefore represent a defense mechanism against this ROS, in addition to SOD activity. This could also explain why neither SOD1 nor SOD2 is increased in F1 *focus* in order to detoxify O_2_^−^.

Previous data obtained in our laboratory [18] showed that SOD1 activity is impaired by 24 h treatment with CdCl_2_, leading to an increased O_2_^−^ level; however, toxicogenomic data showed that the SOD1 coding gene is not dysregulated by CdCl_2_ treatment neither in C3H cells [3] nor in human SH-SY5Y neuroblastoma cells [27]. In *foci*, we could not detect any impairment in SOD1 activity, suggesting that this enzyme inhibition is an early event caused by CdCl_2_ administration, far before the 4 to 6 weeks needed for the in vitro cell transformation. Finally, no autophagy activation was detected in the two *foci*, suggesting that the autophagy observed after cadmium administration for 24 h was part of the early mechanism of defense against this metal. By comparing data reported in this paper with those previously obtained on healthy C3H10T1/2Cl8 cells treated with CdCl_2_ for 24 h [18], we observed that each *focus* maintains some but not all the alterations observed in C3H10T1/2Cl8. This finding suggests that the different alterations did not originally occur in all treated cells, but that different cells reacted to CdCl_2_ exposure in different ways. In other words, although C3H cells are all genetically identical, much of their fate upon CdCl_2_ exposure depends on a plethora of microenvironmental, epigenetic and subcellular factors, such as, for example, local metallothioneins and GST expression and ROS defense enzyme activities.

Although in different ways, both F1 and F3 *foci* showed uncoordinated glycolysis, TCA and oxidative phosphorylation, each pathway working independently. This is likely a consequence of the loss of the Pasteur effect in F1 *focus* and of mitochondrial damage in F3 *focus*, each leading to cell transformation and *foci* formation.

CdCl_2_ toxicity is efficiently inactivated in most cells; however, a very small number of cells are damaged, in different ways. These cells proliferate in the absence of CdCl_2_, giving rise to *foci* after 4 to 6 weeks. Further research will address the question of how all these effects are irreversibly triggered by CdCl_2_ uptake, 4–6 weeks earlier than *foci* detection and collection.

## 4. Materials and Methods

### 4.1. Cell and Culture Conditions

The experiments were performed using the cells collected from cadmium-transformed *foci* obtained at the end of Cell Transformation Assays (CTAs) on C3H10T1/2 clone 8 mouse embryonic fibroblasts (cell line ATCC, CCL 226 lot. n. 58078542), as previously described [16]. This cell line was chosen for its high sensitivity to carcinogenic compounds, its low spontaneous transformation rates, and because it represents one of the three cell lines suggested in the Detailed Review Paper on Cell Transformation Assay to be used for detection of chemical carcinogens [14]. Cells with passages from 9 to 12 were used for cell transformation studies [14]. The CTA was carried out following cells exposure to 1 μM CdCl_2_ for 24 h; after that, the cadmium containing medium was substituted with a fresh medium without cadmium and the cells were grown for 6 weeks, until *foci* appeared [28]. Different *foci* were collected at the end of the CTAs, cultured and processed for further analyses, as described in the following sections. Among the different fully transformed *foci*, F1 and F3 *foci* were subjected to further analyses, since they were found to be very different, both morphologically and from the biochemical point of view. Cells were cultured in Basal Medium Eagle (BME, Sigma Chemical Co., St. Louis, MO, USA) enriched with 10% heat-inactivated fetal bovine serum (FBS, Euroclone, Pero, Italy), 1 % glutamine, 0.5% HEPES 2 M and 25 μg/mL gentamicin (all purchased from Sigma) at 37 °C in a humidified incubator supplied with a constant flow of 5% CO_2_ in air throughout each experiment. Cells were routinely seeded in 100 mm Ø cell culture Petri dishes, the medium was changed every 3 days and cells grown until 80% confluence maximum was reached. The cells were stored in ampoules, frozen at –80 °C with 10% sterile dimethyl sulfoxide (DMSO) as a preservative.

### 4.2. Cell Lysis and Preparation of Protein Samples

Cells from both F1 and F3 *foci* were harvested by trypsinization at 80% confluence, rinsed with ice-cold PBS and lysed in 50 mM Tris-HCl, pH 7.4, 150 mM NaCl, 5 mM EDTA, 10% glycerol, 1% NP40 buffer, containing 1 μM leupeptin, 2 μg/mL aprotinin, 1 μg/mL pepstatin and 1 mM phenylmethylsulfonyl fluoride (PMSF). After lysis on ice, homogenates were obtained by passing the cells 5 times through a blunt 20-gauge needle fitted to a syringe and then centrifuging at 15,000× *g* for 30 min at 4 °C. Enzyme activities were assayed on supernatants.

For cytosolic fraction, cells were rinsed with ice-cold PBS and lysed in PBS, containing 1 μM leupeptin, 2 μg/mL aprotinin, 1 μg/mL pepstatin and 1 mM PMSF; homogenates were obtained by passing the cells 5 times through a blunt 20-gauge needle fitted to a syringe, incubating on ice for 15 min and sonicating 2 times (10 s cycle). The supernatant was obtained by centrifugation at 15,000× *g* for 10 min at 4 °C and used to measure enzyme activities.

For mitochondrial fraction, cell lysate was centrifuged at 800× *g* for 5 min at 4 °C to remove nuclei and unbroken cells. The post-nuclear supernatant was centrifuged at 10,000× *g* for 15 min to collect mitochondria. After centrifugation, the supernatant was removed and pellets were resuspended in 50 mM Tris-HCl 50, pH 7.4, 150 mM NaCl, 5 mM EDTA, 10% glycerol, 1% NP40 buffer, containing 1 μM leupeptin, 2 μg/mL aprotinin, 1 μg/mL pepstatin and 1 mM PMSF and used to measure enzymatic activities. The cytosolic and mitochondrial fractions were analyzed through Western blot to confirm mitochondria isolation.

### 4.3. Cell Lysis and Preparation of Metabolite Samples

Both cell clones were harvested by trypsinization at 80% confluence; the pellets were resuspended in 3 mL PBS, harvested by a centrifugation in the above conditions, weighed, and resuspended with 5 volumes of 5% perchloric acid. The suspension was passed 5 times through a blunt 20-gauge needle fitted to a syringe, incubated on ice for 15 min and centrifuged at 3000× *g* at 4 °C for 10 min. The resulting supernatant was neutralized with 2.5 M K_2_CO_3_ to pH 6.5, centrifuged at 3000× *g* at 4 °C for 10 min to eliminate potassium perchlorate and kept at −80 °C for metabolite analyses.

### 4.4. Enzyme and Metabolite Assays

The enzymes and metabolites were assayed using the following procedures. All assays were performed in triplicate at 30 °C in a Cary3 Spectrophotometer and analyzed by the Cary Win UV application software for Windows. Enzymatic activities were expressed in international units and referred to protein concentration as determined by the Bradford method [29]. Metabolite concentrations were expressed in nmol/mg cells.

Hexokinase (HK) activity was determined by following NADPH formation at 340 nm by coupled assay with glucose-6-phosphate dehydrogenase (G6PDH) according to Bergmeyer [30]. The protein samples were incubated with 100 mM Tris-HCl pH 7.6, 1 mM ATP, 0.6 mM NADP^+^, 2 mM glucose, 10 mM MgCl_2_, 1 U/mL G6PDH.

In glyceraldeyde-3-phosphate dehydrogenase (GAPDH) assay was measured the disappearance of NADH at 340 nm according to Bergmeyer [30]. The protein samples were incubated with 80 mM HEPES-Tris pH 7.6, 6 mM glycerate 3-phosphate, 0.9 mM EDTA, 1.1 mM ATP, 0.2 mM NADH, 1.7 mM MgSO_4_, 15 U/mL phosphoglycerate kinase (PGK). In lactate dehydrogenase (LDH) assay was measured the disappearance of NADH at 340 nm according to Bergmeyer [30]. The protein samples were incubated with 85 mM potassium phosphate buffer, 0.2 mM NADH, 0.6 mM pyruvate. Pyruvate kinase (PK) activity was determined by following NADH disappearance at 340 nm by coupled assay with lactate dehydrogenase (LDH) according to Bergmeyer [30]. The protein samples were incubated with 50 mM HEPES-Tris pH 7.6, 75 mM KCl, 8 mM MgCl2, 0.2 mM NADH, 8 mM 2- phosphoenolpyruvate, 1.5 mM ADP, 9 U/mL LDH. In malate dehydrogenase (MDH) assay was measured the disappearance of NADH at 340 nm according to Bergmeyer [30]. The protein samples were incubated with 50 mM HEPES-Tris pH 7.6, 0.5 mM oxaloacetate, 0.2 mM NADH. In glutamate dehydrogenase (GLDH) assay was measured the disappearance of NADH at 340 nm according to Bergmeyer [30]. The protein samples were incubated with 50 mM HEPES-Tris pH 7.6, 100 mM NH_4_Cl, 0.2 mM NADH, 1 mM ADP, 1 mM EDTA, 7 mM 2-oxoglutarate. Glucose-6-phosphate dehydrogenase (G6PDH) activity was determined by following NADPH formation at 340 nm according to Bergmeyer [30]. The protein samples were incubated with Tris-HCl 90 mM, 7 mM MgCl_2_, 1 mM glucose-6-phosphate, 0.4 mM NADP^+^.

NADP^+^ dependent isocitrate dehydrogenase (ICDH) activity was determined by following NADPH formation at 340 nm according to Bergmeyer [30]. The protein samples were incubated with 50 mM HEPES-Tris pH 7.6, 4 mM MnCl_2_, 3.7 mM isocitrate, 0.32 mM NADP^+^. Malic enzyme (ME) activity was determined by following NADPH formation at 340 nm according to Bergmeyer [30]. The protein samples were incubated with 50 mM HEPES-Tris pH 7.6, 10 mM MgSO_4_, 4 mM malate, 0.5 mM NADP^+^. Citrate synthase (CS) was assayed according to Shepherd [31]. In detail, the protein samples were incubated with 100 mM Tris-HCl pH 8, 0.1 mM 5,5′- dithiobis-(2-nitrobenzoic acid), 0.05 mM acetyl-CoA and 0.25 mM oxaloacetate. The reaction was monitored at 412 nm. Catalase (CAT) activity was assayed according to Bergmeyer [32], using 12 mM H_2_O_2_ as substrate in the presence of 50 mM sodium phosphate buffer, pH 7.5. The reaction was monitored at 240 nm. Glutathione S-transferase (GST) was measured as reported in Habig [33], using 1 mM reduced glutathione (GSH) and 1 mM 1-chloro-2,4-dinitrobenzene (CDNB) as substrates in the presence of 90 mM potassium phosphate buffer pH 6.5. The reaction was monitored at 340 nm. The glutathione peroxidase (GPx) activity is based on the oxidation of GSH using H_2_O_2_ as substrate, coupled to the disappearance of NADPH by glutathione reductase (GR), according to Nakamura [34]. The protein samples were incubated with 50 mM sodium phosphate buffer pH 7.5, 0.16 mM NADPH, 1 mM NaN_3_, 0.4 mM EDTA, 1 mM GSH, 0.2 mM H_2_O_2_, 2 U/mL GR.

Glutathione reductase (GR) was measured following the disappearance of NADPH at 340 nm according to Wang [35]. The protein samples were incubated with 100 mM potassium phosphate buffer pH 7.6, 0.16 mM NADPH, 1 mM EDTA, 1mg/mL BSA, 4.6 mM oxidized glutathione (GSSG). Superoxide dismutase 1 (SOD1) and superoxide dismutase 2 (SOD2) were measured using an indirect method according to Vance [36]. This technique is based on the ability of superoxide dismutase to compete with ferricytochrome c for superoxide anions generated by the xanthine oxidase system and thus to inhibit the reduction of ferricytochrome c. Briefly, the protein samples were incubated with 0.01 mM ferricytochrome c in 10 mM HEPES-Tris pH 7.5, 0.1 mM EDTA, 0.01 mM xanthine in 1 mM NaOH and xanthine oxidase at final concentration of 0.006 U/mL. Under these conditions, one unit of SOD is the amount of enzyme able to yield a 50% decrease in the rate of ferricytochrome c reduction followed at 550 nm.

Lactate and total intracellular ATP were measured using standard enzymatic tests [30]. In detail, lactate was measured following NADH formation in the presence of 380 mM Tris-Glycine pH 9, 2.6 mM NAD^+^, 15 U/mL lactate dehydrogenase; ATP was measured following NADPH formation in the presence of 25 mM HEPES-Tris pH 7.6, 3.3 mM MgCl_2_, 0.23 mM NADP^+^, 17 mM glucose, 0.23 U/mL G6PDH, 0.46 U/mL HK.

### 4.5. GSH Assay

Both *foci* were harvested by trypsinization at 80% confluence; the pellets were washed in 3 mL PBS, harvested by a centrifugation and weighed to normalize the results to mg of cells. Pellets were resuspended in 500 μL cold 5% 5-sulfosalicylic acid (SSA), lysed by vortexing and by passing through a blunt 20-gauge needle fitted to a syringe 5 times. All the samples were incubated for 10 min at 4 °C and then centrifuged at 14,000× *g* for 10 min at 4 °C. The supernatant was prepared and used for the analysis following the instructions of Glutathione Colorimetric Detection Kit (catalog number EIAGSHC, Invitrogen, Carlsbad, CA, USA). The Kit is designed to measure oxidized glutathione (GSSG), total glutathione (GSH tot) and reduced glutathione (GSH tot—GSSG) concentrations through enzymatic recycling assay based on glutathione reductase and reduction of Ellman reagent (5,5-dithiobis(2-nitrobenzoic acid)) and using 2-vinylpyridine as reagent for the derivatization of glutathione [37]. Therefore, it was possible to obtain GSH/GSSG ratio, a critical indicator of cell health. The absorbance was measured at 405 nm using a micro plate reader. The values of absorbance were compared to standard curves (GSH tot and GSSG, respectively) and normalized to mg of cells. Final concentrations were expressed in nmol/mg cells.

### 4.6. SDS-PAGE and Western Blotting

For sample preparation for Western-blot analysis, both cell clones were harvested by trypsinization at 80% confluence. The cells were then rinsed with ice-cold PBS and lysed in RIPA buffer (50 mM Tris-HCl pH 7.5, 150 mM NaCl, 1% NP-40, 0.5% sodium deoxycholate, 0.1% SDS) containing 1 μM leupeptin, 2 μg/mL aprotinin, 1 μg/mL pepstatin, 1 mM PMSF and phosphatase inhibitors. After lysis on ice, homogenates were obtained by passing them through a blunt 20-gauge needle fitted to a syringe 5 times; they were then centrifuged at 15,000× *g* for 30 min. Supernatants were analyzed for protein content by the BCA protein assay [38]. SDS-PAGE and Western blot were carried out by standard procedures [39]. Sixty micrograms of proteins were separated on a 10% or 15% acrylamide/bis-acrylamide SDS-PAGE, transferred onto a nitrocellulose membrane (Millipore, Billerica, MA, USA), probed with the appropriated antibodies and visualized using ECL detection system (Millipore). Protein levels were quantified by densitometry of immunoblots using Scion Image software (Scion Corp., Frederick, MD, USA). The following primary antibodies were used (all purchased by Cell Signaling Technology, Danvers, MA, USA): anti-PKM2 (catalog number #4053, dilution 1:1000), anti-PFKFB3 (catalog number #13123, dilution 1:1000), anti P-AMPK (Thr172) (catalog number #2535, dilution 1:1000), anti-AMPK (catalog number #2532, dilution 1:1000), anti- LC3B (catalog number #2775, dilution 1:500), anti-GAPDH (catalog number #2118, dilution 1:10,000), anti-tubulin (catalog number #2125S, dilution 1:1000) (all purchased by Cell Signaling Technology, Danvers, MA, USA) and anti-vinculin (catalog number #V9131, dilution 1:10,000) (purchased by Sigma Chemical Co., St. Louis, MO, USA). IgG HRP anti-rabbit (catalog number #7074) and anti-mouse (catalog number #7076) conjugated secondary antibodies (purchased by Cell Signaling Technology, Danvers, MA, USA) were diluted 1:10,000.

### 4.7. Detection of Intracellular Reactive Oxygen Species

The generation of intracellular reactive oxygen species (ROS) was detected by the oxidation of 2′,7′-Dichlorofluorescin diacetate (H_2_DCFDA) or Dihydroethidium (DHE). H_2_DCFDA is an indicator for both reactive oxygen species and nitric oxide (•NO); the second probe is more selective towards superoxide anion (O_2_^−^). The cells were plated at a density of 2.5 × 10^5^ cells per well into six-well plates in 2 mL of complete culture medium and incubated 24 h after seeding with H_2_DCFDA (5 μΜ final concentration in PBS) or DHE (10 μΜ final concentration in complete medium) for 20 min in the dark at 37 °C. At the end of incubation, cells were washed by warm PBS, trypsinized (500 μL of trypsin/well) and harvested by centrifugation (5 min at 2000× *g*) at room temperature. The pellet was resuspended in 500 μL/tube of PBS and ROS generation of 10,000 cells was measured by the fluorescence intensity. FL-1 channel (530 nm) was utilized to detect the fluorescence intensity of DCF; HE fluorescence can be measured at 585 nm, or FL-2 channel, band-pass filter. Logarithmic amplification, which produces an output signal proportional to the logarithm of the input signal, was used to detect probe fluorescence. Data quality is enhanced when the brightness levels of all probes excited off a single laser are balanced within one log scale of fluorescence intensity [40]. Flow cytometry data were analyzed using CytExpert 2.3 Software (Beckman Coulter, Inc., Brea, CA, USA).

### 4.8. Detection of Mitochondrial ROS (mtROS)

MitoSOX ™ Red (ThermoFisher Scientific, Massachusetts, USA) and MitoPY1 (Tocris Bioscience, Bristol, UK) indicators were used to detect the mitochondrial superoxide anion and mitochondrial hydrogen peroxide production, respectively, in intact adherent cells. The oxidation of these probes forms intermediate probe-derived radicals that are successively oxidized to generate the corresponding fluorescent products [41]. The cells were plated at a density of 2.5 × 10^5^ cells per well into six-well plates in 2 mL of complete culture medium and stained 24 h after seeding with MitoSOX™ Red or MitoPY1 (5 μΜ final concentration in 1 mL of PBS) for 20 min in the dark at 37 °C. After staining, the cells were washed by warm PBS, trypsinized (500 μL of trypsin /well) and harvested by centrifugation (5 min at 2000× *g*) at room temperature. The pellet was resuspended in 500 μL/tube of PBS and mtROS generation of 10,000 cells was measured by the fluorescence intensity. FL-1 channel (530 nm) was utilized to detect the fluorescence intensity of MitoPY1; FL-2 channel (585 nm) band-pass filter was utilized to measure the fluorescence intensity of MitoSOX ™ Red. Logarithmic amplification, which produces an output signal proportional to the logarithm of the input signal, was used to detect probe fluorescence. Data quality is enhanced when the brightness levels of all probes excited off a single laser are balanced within one log scale of fluorescence intensity [40]. Flow cytometry data were analyzed using CytExpert 2.3 Software (Beckman Coulter, Inc., Brea, CA, USA).

### 4.9. Mitochondrial Transmembrane Potential (MTP) Assay

MTP alterations were assessed by flow cytometry, using the mitochondrial potential sensitive dye 3,3′-dihexyloxacarbocyanine Iodide (DiOC6), which accumulates in mitochondria due to their negative membrane potential and can be applied to monitor the mitochondrial membrane potential using flow cytometry detection. The cells were plated at a density of 2.5 × 10^5^ cells per well into six-well plates in 2 mL of complete culture medium, harvested 24 h after seeding by centrifugation (5 min at 2000× *g*) at room temperature and stained with DiOC6 (40 nM in PBS, 20 min at 37 °C and 5% CO2 in the dark). Loss in DiOC6 fluorescence indicates disruption of the mitochondrial inner transmembrane potential. The probe was excited at 488 nm and emission was measured through a 530 nm (FL-1 channel) band-pass filter. Logarithmic amplification, which produces an output signal proportional to the logarithm of the input signal, was used to detect probe fluorescence. Data quality is enhanced when the brightness levels of all probes excited off a single laser are balanced within one log scale of fluorescence intensity [40]. Flow cytometry data were analyzed using CytExpert 2.3 Software (Beckman Coulter, Inc., Brea, CA, USA).

### 4.10. Oxygen Consumption Rate and Extra-Cellular Acidification Rate Measurements

Oxygen consumption rate (OCR) and extra-cellular acidification rate (ECAR) were measured in F1 and F3 *foci* with Seahorse XFe24 Analyzer (Seahorse Bioscience, Billerica, MA, USA) using Seahorse XF Cell Mito Stress Test Kit (catalog number #103015-100, purchased by Agilent Technologies, Santa Clara, CA, USA) and Agilent Seahorse XF Glycolytic Rate Assay Kit (catalog number #103344-100, purchased by Agilent Technologies, Santa Clara, CA, USA). The cells were seeded in Agilent Seahorse XF24 cell culture microplates at density of 3 × 10^4^ cells/well in 250 μL of Basal Medium Eagle and 24 h after seeding the growth medium was replaced with 525 μL/well of Seahorse XF Base Medium containing 1 mM pyruvate, 2 mM glutamine and 10 mM glucose for the Cell Mito Stress Test Kit or 1 mM pyruvate, 2 mM glutamine, 10 mM glucose and 5 mM HEPES for the Glycolytic Rate Assay Kit. Then, the plate was incubated into 37 °C non-CO_2_ incubator for 1 h, before starting the experiment procedure.

The sensor cartridge was calibrated by Seahorse XFe24 Analyzer. Pre-warmed oligomycin, FCCP, rotenone and antimycin A were loaded into injector ports A, B and C of sensor cartridge, to reach working concentration of 1 μM, 2 μM and 0.5 μM, respectively, for the Cell Mito Stress Test Kit.

Pre-warmed rotenone and antimycin A and 2-deoxy-D-glucose (2-DG) were loaded into injector ports A and B of sensor cartridge, to reach working concentration of 0.5 μM and 50 mM for the Glycolytic Rate Assay Kit.

OCR and ECAR were detected under basal conditions followed by the sequential addition of the drugs, to measure non-mitochondrial respiration, maximal respiration, proton leak, ATP respiration, respiratory capacity, coupling efficiency for the Cell Mito Stress Test Kit and basal glycolysis, basal proton efflux rate, compensatory glycolysis and post 2-DG acidification for the Glycolytic Rate Assay Kit [42,43,44]. After assay performance, cells were lysed and the total cellular proteins were quantified using Bradford method [29] in order to normalize the data.

### 4.11. Confocal Microscopy

Mitochondria fluorescence was studied by laser scanning confocal microscopy, using a Bio-Rad MRC-600 confocal microscope (Bio-Rad, Hemel Hempstead, UK) equipped with a 25 mW argon laser. The scanning head was coupled with an upright epifluorescence microscope Nikon Optiphot-2 (Nikon, Tokyo, Japan) with a 60× oil immersion objective Nikon Planapochromat (N.A. = 1.4). The fluorescence was excited at 488 nm and the emission was collected through a long pass filter above 515 nm. High sensitivity photon counting detection was used to minimize the excitation power (0.1 mW at the entry of the optical head) and preserve cell viability.

The cells were plated in 35 mm Ø Petri dishes at density of 6 × 10^4^ cells/well and 24 h after seeding the medium was removed, the cells were washed twice with phosphate buffer saline (PBS) and incubated for 10 min in 1 μM rhodamine 123 (R123) solution at 37 °C and 5% CO_2_. After incubation, the cells were rinsed twice with PBS and few microliters of PBS were left in the Petri dish to avoid cell drying. A coverslip was placed over the cells that were immediately imaged by confocal microscope.

To measure mitochondrial interconnectivity and elongation from confocal microscope images we used the macro designed by Dagda and colleagues [45].

### 4.12. Statistical Analysis

The data were tested by Student’s *t*-test. All calculations were conducted using the R statistics programming environment [46].

## Figures and Tables

**Table 1:** Glycolytic metabolism and Krebs cycle enzymes activities in F1 and F3 *foci*. Results are expressed as U/mg and are shown as mean ± SEM obtained in three independent experiments. Statistical significance (F1 vs. F3): * *p* < 0.05 (Student’s *t*-test).

Enzyme	F1	F3	*p*-Value
	U/mg	U/mg	
Hexokinase	0.028 ± 0.003	0.017 ± 0.002	*
Glyceraldehyde-3-phosphate dehydrogenase	0.187 ± 0.041	0.049 ± 0.025	*
Pyruvate kinase	0.031 ± 0.001	0.028 ± 0.001	*
Citrate synthase	0.109 ± 0,009	0.087 ± 0.007	*
Malate dehydrogenase	0.296 ± 0.038	0.175 ± 0.022	*
Glutamate dehydrogenase	0.057 ± 0.003	0.068 ± 0.004	*
Malic enzyme	0.012 ± 0.001	0.014 ± 0.0001	
Glucose-6-phosphate dehydrogenase	0.012 ± 0.001	0.011 ± 0.0009	
Isocitrate dehydrogenase	0.031 ± 0.005	0.025 ± 0.004	

**Table 2:** Enzymes involved in oxidative stress defense in F1 and F3 *foci*. Results are expressed as U/mg and are shown as mean ± SEM obtained in three independent experiments. Statistical significance (F1 vs. F3): * *p* < 0.05, ** *p* < 0.01 (Student’s *t*-test).

Enzyme	F1	F3	*p*-Value
	U/mg	U/mg	
Glutathione peroxidase	0.148 ± 0.015	0.211 ± 0.021	*
Glutathione reductase	0.016 ± 0.0005	0.012 ± 0.001	*
Glutathione S-transferase	0.066 ± 0.008	0.035 ± 0.007	*
Catalase	4.788 ± 0.594	7.354 ± 0.442	**
Superoxide dismutase 1	0.591 ± 0.050	0.559 ± 0.073	
Superoxide dismutase 2	1.750 ± 0.170	1.240 ± 0.150	

**Table 3:** Confocal images of F1 and F3 *foci* analysis. Results are shown as mean ± SEM obtained analyzing 30 different cells for each *focus*.

Parameters	F1	F3
**Circularity**	0.78 ± 0.007	0.75 ± 0.008
**Density of mitochondrial network**	0.18 ± 0.003	0.16 ± 0.004
